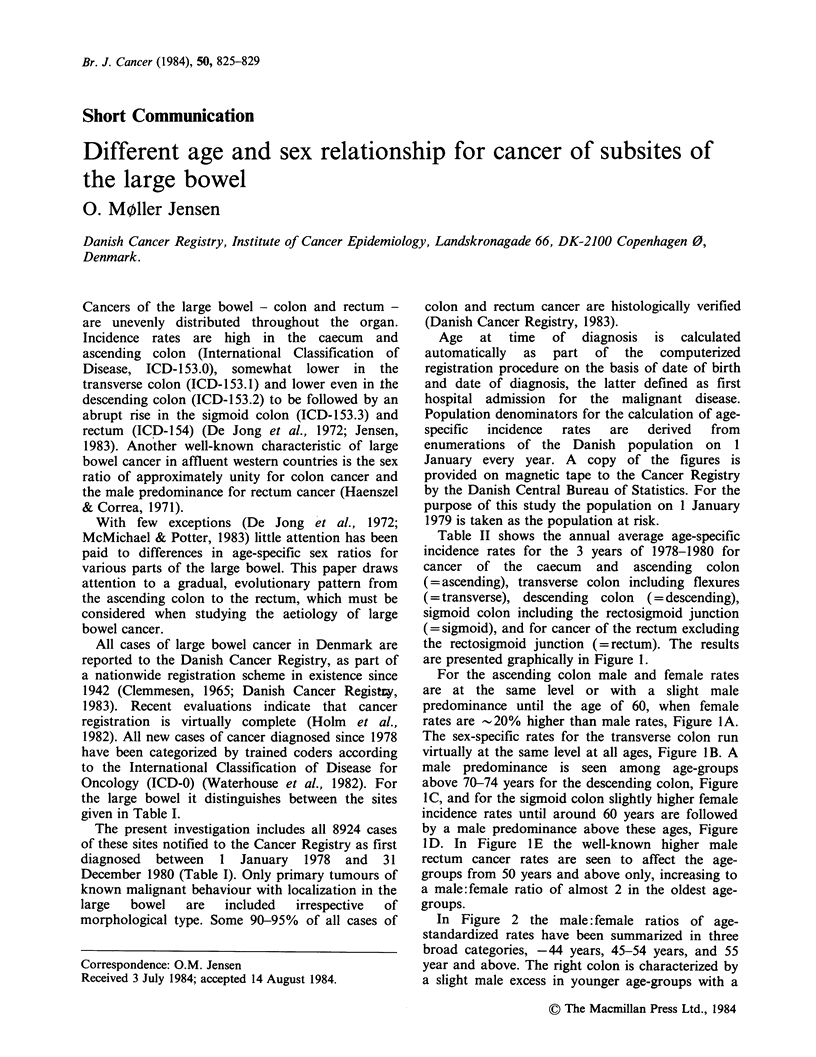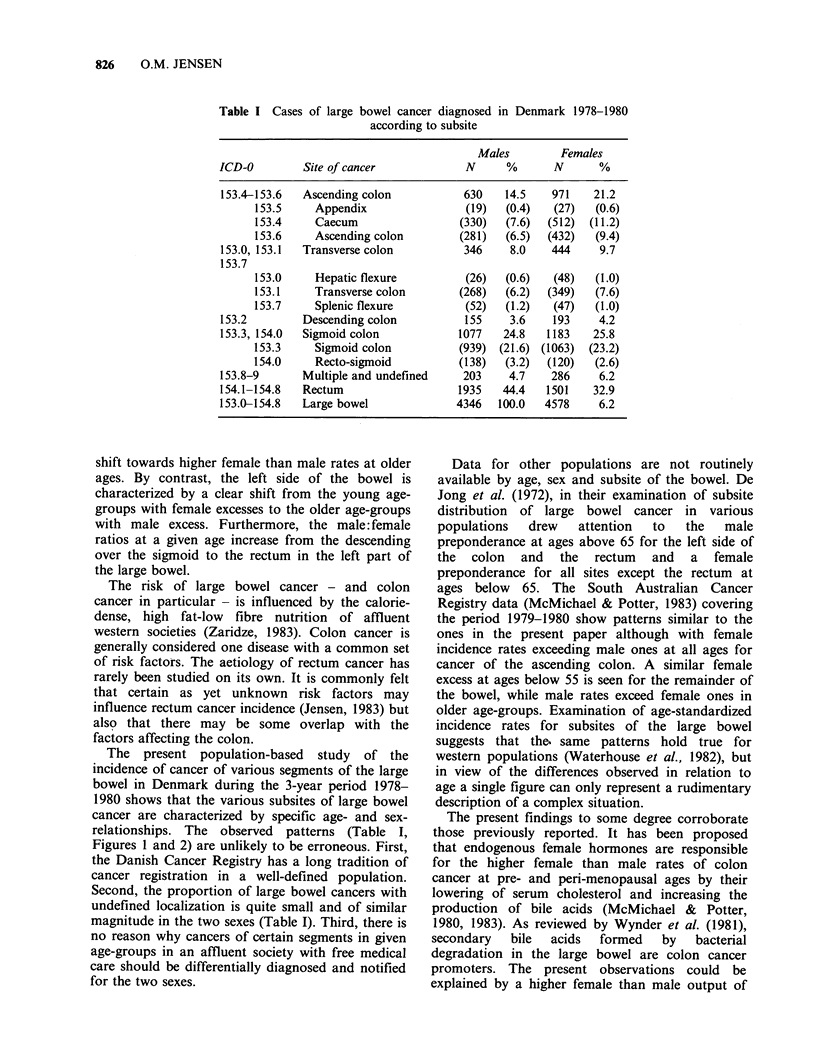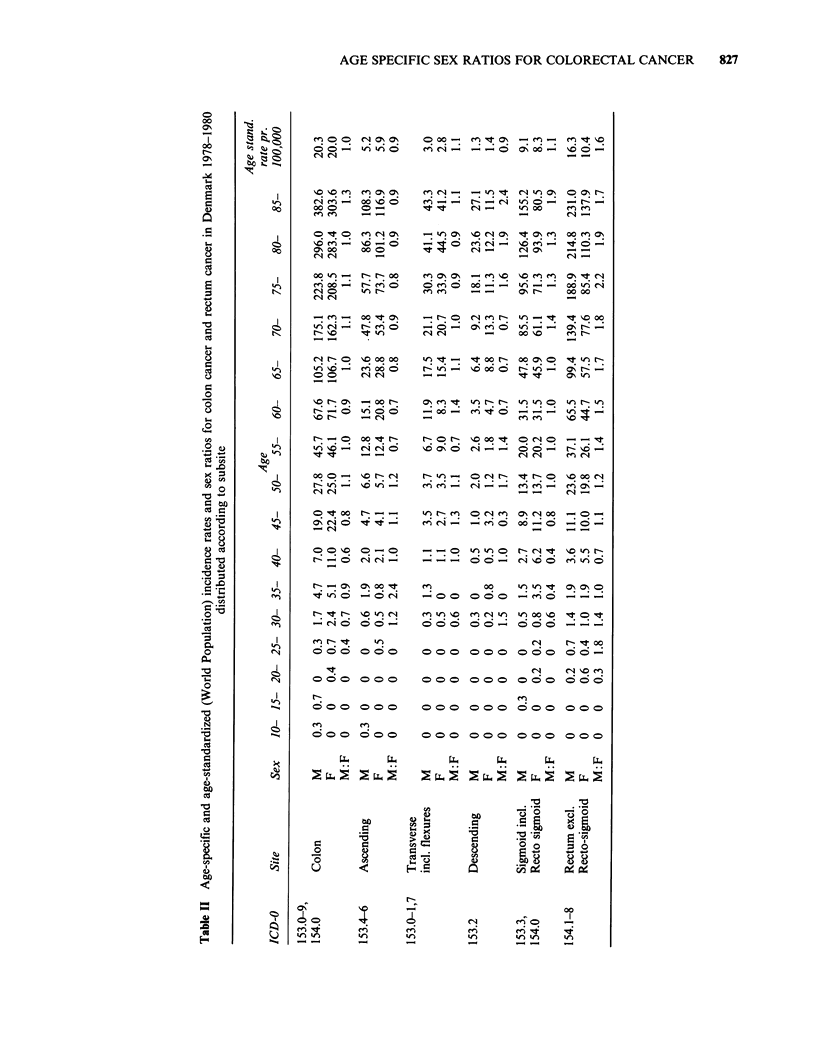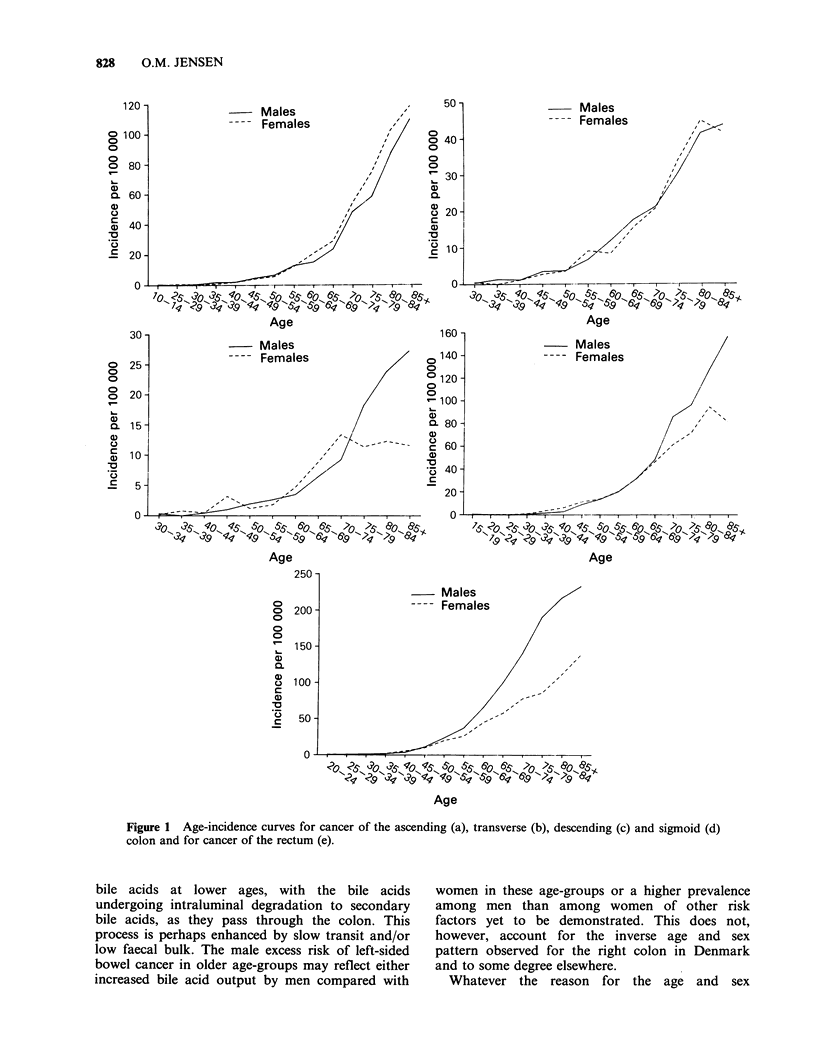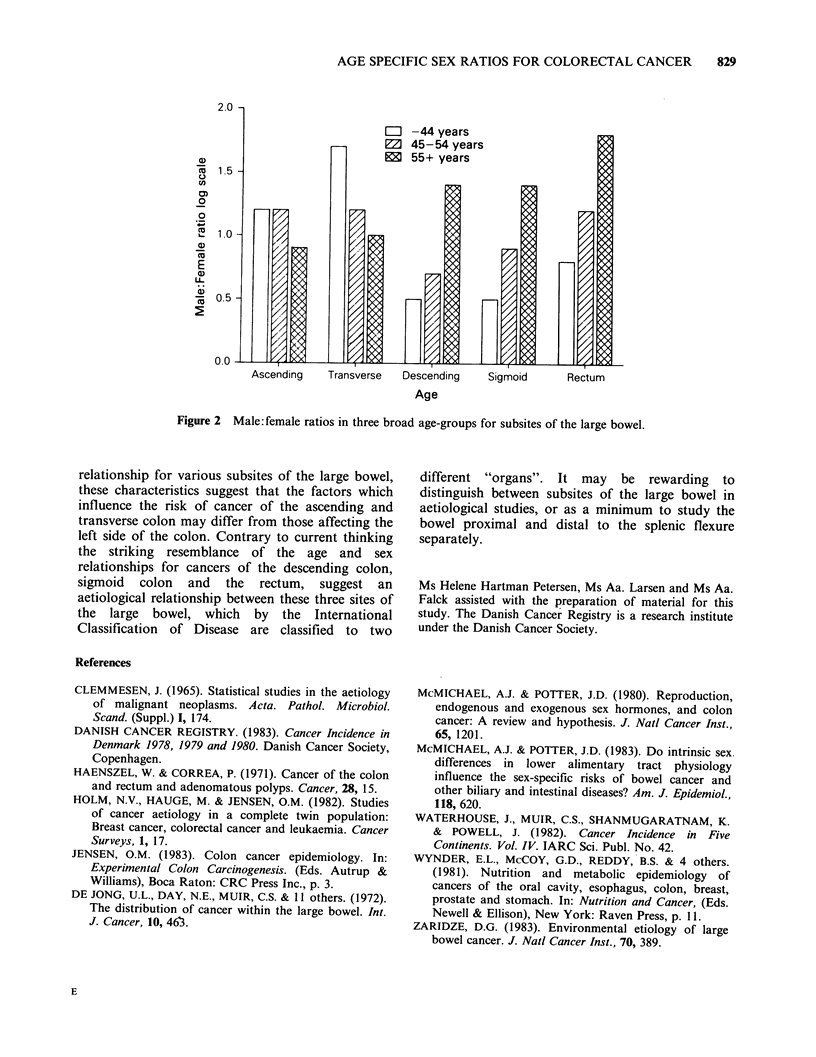# Different age and sex relationship for cancer of subsites of the large bowel.

**DOI:** 10.1038/bjc.1984.262

**Published:** 1984-12

**Authors:** O. M. Jensen


Br. J. Cancer (1984), 50, 825–829

### Short Communication

### Different age and sex relationship for cancer of subsites of the large bowel

O. Møller Jensen

*Danish Cancer Registry, Institute of Cancer Epidemiology, Landskronagade 66, DK-2100 Copenhagen Ø, Denmark.*

Cancers of the large bowel – colon and rectum – are unevenly distributed throughout the organ. Incidence rates are high in the caecum and ascending colon (International Classification of Disease, ICD-153.0), somewhat lower in the transverse colon (ICD-153.1) and lower even in the descending colon (ICD-153.2) to be followed by an abrupt rise in the sigmoid colon (ICD-153.3) and rectum (ICD-154) (De Jong *et al.*, 1972; Jensen, 1983). Another well-known characteristic of large bowel cancer in affluent western countries is the sex ratio of approximately unity for colon cancer and the male predominance for rectum cancer (Haenszel & Correa, 1971).

With few exceptions (De Jong *et al.*, 1972; McMichael & Potter, 1983) little attention has been paid to differences in age-specific sex ratios for various parts of the large bowel. This paper draws attention to a gradual, evolutionary pattern from the ascending colon to the rectum, which must be considered when studying the aetiology of large bowel cancer.

All cases of large bowel cancer in Denmark are reported to the Danish Cancer Registry, as part of a nationwide registration scheme in existence since 1942 (Clemmesen, 1965; Danish Cancer Registry, 1983). Recent evaluations indicate that cancer registration is virtually complete (Holm *et al.*, 1982). All new cases of cancer diagnosed since 1978 have been categorized by trained coders according to the International Classification of Disease for Oncology (ICD-0) (Waterhouse *et al.*, 1982). For the large bowel it distinguishes between the sites given in Table I.

The present investigation includes all 8924 cases of these sites notified to the Cancer Registry as first diagnosed between 1 January 1978 and 31 December 1980 (Table I). Only primary tumours of known malignant behaviour with localization in the large bowel are included irrespective of morphological type. Some 90–95% of all cases of colon and rectum cancer are histologically verified (Danish Cancer Registry, 1983).

Age at time of diagnosis is calculated automatically as part of the computerized registration procedure on the basis of date of birth and date of diagnosis, the latter defined as first hospital admission for the malignant disease. Population denominators for the calculation of age-specific incidence rates are derived from enumerations of the Danish population on 1 January every year. A copy of the figures is provided on magnetic tape to the Cancer Registry by the Danish Central Bureau of Statistics. For the purpose of this study the population on 1 January 1979 is taken as the population at risk.

Table II shows the annual average age-specific incidence rates for the 3 years of 1978–1980 for cancer of the caecum and ascending colon (= ascending), transverse colon including flexures (= transverse), descending colon (= descending), sigmoid colon including the rectosigmoid junction (= sigmoid), and for cancer of the rectum excluding the rectosigmoid junction (= rectum). The results are presented graphically in Figure 1.

For the ascending colon male and female rates are at the same level or with a slight male predominance until the age of 60, when female rates are ~20% higher than male rates, Figure 1A. The sex-specific rates for the transverse colon run virtually at the same level at all ages, Figure 1B. A male predominance is seen among age-groups above 70–74 years for the descending colon, Figure 1C, and for the sigmoid colon slightly higher female incidence rates until around 60 years are followed by a male predominance above these ages, Figure 1D. In Figure 1E the well-known higher male rectum cancer rates are seen to affect the age-groups from 50 years and above only, increasing to a male:female ratio of almost 2 in the oldest age-groups.

In Figure 2 the male:female ratios of age-standardized rates have been summarized in three broad categories, −44 years, 45–54 years, and 55 year and above. The right colon is characterized by a slight male excess in younger age-groups with a

Correspondence: O.M. Jensen
Received 3 July 1984; accepted 14 August 1984.

© The Macmillan Press Ltd., 1984

**Table I** Cases of large bowel cancer diagnosed in Denmark 1978–1980 according to subsite

| | | Males | | Females | |
|---|---|---|---|---|---|
| *ICD-0* | *Site of cancer* | *N* | % | *N* | % |
| 153.4–153.6 | Ascending colon | 630 | 14.5 | 971 | 21.2 |
| 153.5 | Appendix | (19) | (0.4) | (27) | (0.6) |
| 153.4 | Caecum | (330) | (7.6) | (512) | (11.2) |
| 153.6 | Ascending colon | (281) | (6.5) | (432) | (9.4) |
| 153.0, 153.1 153.7 | Transverse colon | 346 | 8.0 | 444 | 9.7 |
| 153.0 | Hepatic flexure | (26) | (0.6) | (48) | (1.0) |
| 153.1 | Transverse colon | (268) | (6.2) | (349) | (7.6) |
| 153.7 | Splenic flexure | (52) | (1.2) | (47) | (1.0) |
| 153.2 | Descending colon | 155 | 3.6 | 193 | 4.2 |
| 153.3, 154.0 | Sigmoid colon | 1077 | 24.8 | 1183 | 25.8 |
| 153.3 | Sigmoid colon | (939) | (21.6) | (1063) | (23.2) |
| 154.0 | Recto-sigmoid | (138) | (3.2) | (120) | (2.6) |
| 153.8–9 | Multiple and undefined | 203 | 4.7 | 286 | 6.2 |
| 154.1–154.8 | Rectum | 1935 | 44.4 | 1501 | 32.9 |
| 153.0–154.8 | Large bowel | 4346 | 100.0 | 4578 | 6.2 |

shift towards higher female than male rates at older ages. By contrast, the left side of the bowel is characterized by a clear shift from the young age-groups with female excesses to the older age-groups with male excess. Furthermore, the male:female ratios at a given age increase from the descending over the sigmoid to the rectum in the left part of the large bowel.

The risk of large bowel cancer – and colon cancer in particular – is influenced by the calorie-dense, high fat-low fibre nutrition of affluent western societies (Zaridze, 1983). Colon cancer is generally considered one disease with a common set of risk factors. The aetiology of rectum cancer has rarely been studied on its own. It is commonly felt that certain as yet unknown risk factors may influence rectum cancer incidence (Jensen, 1983) but also that there may be some overlap with the factors affecting the colon.

The present population-based study of the incidence of cancer of various segments of the large bowel in Denmark during the 3-year period 1978–1980 shows that the various subsites of large bowel cancer are characterized by specific age- and sex-relationships. The observed patterns (Table I, Figures 1 and 2) are unlikely to be erroneous. First, the Danish Cancer Registry has a long tradition of cancer registration in a well-defined population. Second, the proportion of large bowel cancers with undefined localization is quite small and of similar magnitude in the two sexes (Table I). Third, there is no reason why cancers of certain segments in given age-groups in an affluent society with free medical care should be differentially diagnosed and notified for the two sexes.

Data for other populations are not routinely available by age, sex and subsite of the bowel. De Jong *et al.* (1972), in their examination of subsite distribution of large bowel cancer in various populations drew attention to the male preponderance at ages above 65 for the left side of the colon and the rectum and a female preponderance for all sites except the rectum at ages below 65. The South Australian Cancer Registry data (McMichael & Potter, 1983) covering the period 1979–1980 show patterns similar to the ones in the present paper although with female incidence rates exceeding male ones at all ages for cancer of the ascending colon. A similar female excess at ages below 55 is seen for the remainder of the bowel, while male rates exceed female ones in older age-groups. Examination of age-standardized incidence rates for subsites of the large bowel suggests that the same patterns hold true for western populations (Waterhouse *et al.*, 1982), but in view of the differences observed in relation to age a single figure can only represent a rudimentary description of a complex situation.

The present findings to some degree corroborate those previously reported. It has been proposed that endogenous female hormones are responsible for the higher female than male rates of colon cancer at pre- and peri-menopausal ages by their lowering of serum cholesterol and increasing the production of bile acids (McMichael & Potter, 1980, 1983). As reviewed by Wynder *et al.* (1981), secondary bile acids formed by bacterial degradation in the large bowel are colon cancer promoters. The present observations could be explained by a higher female than male output of

**Table II** Age-specific and age-standardized (World Population) incidence rates and sex ratios for colon cancer and rectum cancer in Denmark 1978–1980 distributed according to subsite

| ICD-0 | Site | Sex | 10– | 15– | 20– | 25– | 30– | 35– | 40– | 45– | 50– | 55– | 60– | 65– | 70– | 75– | 80– | 85– | Age stand. rate pr. 100,000 |
|---|---|---|---|---|---|---|---|---|---|---|---|---|---|---|---|---|---|---|---|
| 153.0–9, 154.0 | Colon | M | 0.3 | 0.7 | 0 | 0.3 | 1.7 | 4.7 | 7.0 | 19.0 | 27.8 | 45.7 | 67.6 | 105.2 | 175.1 | 223.8 | 296.0 | 382.6 | 20.3 |
| | | F | 0 | 0 | 0.4 | 0.7 | 2.4 | 5.1 | 11.0 | 22.4 | 25.0 | 46.1 | 71.7 | 106.7 | 162.3 | 208.5 | 283.4 | 303.6 | 20.0 |
| | | M:F | 0 | 0 | 0 | 0.4 | 0.7 | 0.9 | 0.6 | 0.8 | 1.1 | 1.0 | 0.9 | 1.0 | 1.1 | 1.1 | 1.0 | 1.3 | 1.0 |
| 153.4–6 | Ascending | M | 0.3 | 0 | 0 | 0 | 0.6 | 1.9 | 2.0 | 4.7 | 6.6 | 12.8 | 15.1 | 23.6 | 47.8 | 57.7 | 86.3 | 108.3 | 5.2 |
| | | F | 0 | 0 | 0 | 0.5 | 0.5 | 0.8 | 2.1 | 4.1 | 5.7 | 12.4 | 20.8 | 28.8 | 53.4 | 73.7 | 101.2 | 116.9 | 5.9 |
| | | M:F | 0 | 0 | 0 | 0 | 1.2 | 2.4 | 1.0 | 1.1 | 1.2 | 0.7 | 0.7 | 0.8 | 0.9 | 0.8 | 0.9 | 0.9 | 0.9 |
| 153.0–1,7 | Transverse incl. flexures | M | 0 | 0 | 0 | 0 | 0.3 | 1.3 | 1.1 | 3.5 | 3.7 | 6.7 | 11.9 | 17.5 | 21.1 | 30.3 | 41.1 | 43.3 | 3.0 |
| | | F | 0 | 0 | 0 | 0 | 0.5 | 0 | 1.1 | 2.7 | 3.5 | 9.0 | 8.3 | 15.4 | 20.7 | 33.9 | 44.5 | 41.2 | 2.8 |
| | | M:F | 0 | 0 | 0 | 0 | 0.6 | 0 | 1.0 | 1.3 | 1.1 | 0.7 | 1.4 | 1.1 | 1.0 | 0.9 | 0.9 | 1.1 | 1.1 |
| 153.2 | Descending | M | 0 | 0 | 0 | 0 | 0.3 | 0 | 0.5 | 1.0 | 2.0 | 2.6 | 3.5 | 6.4 | 9.2 | 18.1 | 23.6 | 27.1 | 1.3 |
| | | F | 0 | 0 | 0 | 0 | 0.2 | 0.8 | 0.5 | 3.2 | 1.2 | 1.8 | 4.7 | 8.8 | 13.3 | 11.3 | 12.2 | 11.5 | 1.4 |
| | | M:F | 0 | 0 | 0 | 0 | 1.5 | 0 | 1.0 | 0.3 | 1.7 | 1.4 | 0.7 | 0.7 | 0.7 | 1.6 | 1.9 | 2.4 | 0.9 |
| 153.3, 154.0 | Sigmoid incl. Recto sigmoid | M | 0 | 0.3 | 0 | 0 | 0.5 | 1.5 | 2.7 | 8.9 | 13.4 | 20.0 | 31.5 | 47.8 | 85.5 | 95.6 | 126.4 | 155.2 | 9.1 |
| | | F | 0 | 0 | 0.2 | 0.2 | 0.8 | 3.5 | 6.2 | 11.2 | 13.7 | 20.2 | 31.5 | 45.9 | 61.1 | 71.3 | 93.9 | 80.5 | 8.3 |
| | | M:F | 0 | 0 | 0 | 0 | 0.6 | 0.4 | 0.4 | 0.8 | 1.0 | 1.0 | 1.0 | 1.0 | 1.4 | 1.3 | 1.3 | 1.9 | 1.1 |
| 154.1–8 | Rectum excl. Recto-sigmoid | M | 0 | 0 | 0.2 | 0.7 | 1.4 | 1.9 | 3.6 | 11.1 | 23.6 | 37.1 | 65.5 | 99.4 | 139.4 | 188.9 | 214.8 | 231.0 | 16.3 |
| | | F | 0 | 0 | 0.6 | 0.4 | 1.0 | 1.9 | 5.5 | 10.0 | 19.8 | 26.1 | 44.7 | 57.5 | 77.6 | 85.4 | 110.3 | 137.9 | 10.4 |
| | | M:F | 0 | 0 | 0.3 | 1.8 | 1.4 | 1.0 | 0.7 | 1.1 | 1.2 | 1.4 | 1.5 | 1.7 | 1.8 | 2.2 | 1.9 | 1.7 | 1.6 |

**Figure 1** Age-incidence curves for cancer of the ascending (a), transverse (b), descending (c) and sigmoid (d) colon and for cancer of the rectum (e).

bile acids at lower ages, with the bile acids undergoing intraluminal degradation to secondary bile acids, as they pass through the colon. This process is perhaps enhanced by slow transit and/or low faecal bulk. The male excess risk of left-sided bowel cancer in older age-groups may reflect either increased bile acid output by men compared with women in these age-groups or a higher prevalence among men than among women of other risk factors yet to be demonstrated. This does not, however, account for the inverse age and sex pattern observed for the right colon in Denmark and to some degree elsewhere.

Whatever the reason for the age and sex

**Figure 2** Male:female ratios in three broad age-groups for subsites of the large bowel.

relationship for various subsites of the large bowel, these characteristics suggest that the factors which influence the risk of cancer of the ascending and transverse colon may differ from those affecting the left side of the colon. Contrary to current thinking the striking resemblance of the age and sex relationships for cancers of the descending colon, sigmoid colon and the rectum, suggest an aetiological relationship between these three sites of the large bowel, which by the International Classification of Disease are classified to two different "organs". It may be rewarding to distinguish between subsites of the large bowel in aetiological studies, or as a minimum to study the bowel proximal and distal to the splenic flexure separately.

Ms Helene Hartman Petersen, Ms Aa. Larsen and Ms Aa. Falck assisted with the preparation of material for this study. The Danish Cancer Registry is a research institute under the Danish Cancer Society.

### References

CLEMMESEN, J. (1965). Statistical studies in the aetiology of malignant neoplasms. *Acta. Pathol. Microbiol. Scand.* (Suppl.) **I**, 174.

DANISH CANCER REGISTRY. (1983). *Cancer Incidence in Denmark 1978, 1979 and 1980.* Danish Cancer Society, Copenhagen.

HAENSZEL, W. & CORREA, P. (1971). Cancer of the colon and rectum and adenomatous polyps. *Cancer,* **28,** 15.

HOLM, N.V., HAUGE, M. & JENSEN, O.M. (1982). Studies of cancer aetiology in a complete twin population: Breast cancer, colorectal cancer and leukaemia. *Cancer Surveys,* **1,** 17.

JENSEN, O.M. (1983). Colon cancer epidemiology. In: *Experimental Colon Carcinogenesis.* (Eds. Autrup & Williams), Boca Raton: CRC Press Inc., p. 3.

DE JONG, U.L., DAY, N.E., MUIR, C.S. & 11 others. (1972). The distribution of cancer within the large bowel. *Int. J. Cancer,* **10,** 463.

McMICHAEL, A.J. & POTTER, J.D. (1980). Reproduction, endogenous and exogenous sex hormones, and colon cancer: A review and hypothesis. *J. Natl Cancer Inst.,* **65,** 1201.

McMICHAEL, A.J. & POTTER, J.D. (1983). Do intrinsic sex differences in lower alimentary tract physiology influence the sex-specific risks of bowel cancer and other biliary and intestinal diseases? *Am. J. Epidemiol.,* **118,** 620.

WATERHOUSE, J., MUIR, C.S., SHANMUGARATNAM, K. & POWELL, J. (1982). *Cancer Incidence in Five Continents. Vol. IV.* IARC Sci. Publ. No. 42.

WYNDER, E.L., McCOY, G.D., REDDY, B.S. & 4 others. (1981). Nutrition and metabolic epidemiology of cancers of the oral cavity, esophagus, colon, breast, prostate and stomach. In: *Nutrition and Cancer,* (Eds. Newell & Ellison), New York: Raven Press, p. 11.

ZARIDZE, D.G. (1983). Environmental etiology of large bowel cancer. *J. Natl Cancer Inst.,* **70,** 389.